# Supplementary material for: Thin-film lithium niobate terahertz differential field detectors with a bandwidth reaching 3 terahertz
Source: Nat Commun. 2025 Oct 6;16:8864. doi: 10.1038/s41467-025-63920-2 (PMC12500990; doi:10.1038/s41467-025-63920-2)
Supplement: Supplementary file 1 — Supplementary Information [file 41467_2025_63920_MOESM1_ESM.pdf]

Supplementary Information for

Thin-film lithium niobate terahertz differential field detectors with a bandwidth reaching 3 terahertz

A. Herter, A Shams-Ansari, M. Lončar, J. Faist

1 Supplementary Note 1 (Theory)

1.1 Signal derivation

1.1.1 Interaction inside the antenna gap

For a incoming free-space THz spectrum $E_{\text{THz}}(\Omega)$, the temporal field inside the antenna gab $E_{\text{gap}}(\vec{r},t)$ is determined by the spectral field enhancement of the antenna $G(\Omega)$ and the spatial mode profile inside the antenna gap $u_{\text{THz}}(\vec{r}_\perp)$:

$$E_{\text{gap}}(\vec{r},t) = u_{\text{THz}}(\vec{r}_\perp) \int \mathrm{d}\Omega\, G(\Omega) E_{\text{THz}}(\Omega)\, \mathrm{e}^{-i\Omega t} \tag{1}$$

$$E_{\text{gap}}(\vec{r},t) = u_{\text{THz}}(\vec{r}_\perp) \int \mathrm{d}\Omega\, \mathcal{E}_{\text{gap}}(\Omega)\, \mathrm{e}^{-i\Omega t}. \tag{2}$$

The spatial field $u_{\text{THz}}(\vec{r}_\perp)$ is simulated in CST (details in 2.2). The near-infrared probe field $E_{\text{p}}(\vec{r},t)$ propagating in y-direction inside the lithium niobate waveguide can be described as:

$$E_{\text{p}}(\vec{r},t) = \int \mathrm{d}\omega E_{\text{p}}(\vec{r},\omega) \mathrm{e}^{-i\omega t} \tag{3}$$

$$= u_{\text{p}}(\vec{r}_\perp) \int \mathrm{d}\omega \mathcal{E}_{\text{p}}(\omega) \mathrm{e}^{-i\omega t} \mathrm{e}^{ik(\omega)y}. \tag{4}$$

The spatial profile u_{p} normalized to $\int \mathrm{d}^2 r_\perp |u_{\text{p}}(\vec{r}_\perp)|^2 = S_{\text{eff,p}}$ is independent on the position in propagation direction y and is simulated in section 2.2. We neglect any kind of propagation loss inside the waveguide and the antenna gap in this description, since thin-film lithium niobate provides low-loss waveguiding. $\mathcal{E}_{\text{p}}(\omega)$ describes the spectral field amplitude of the probe field and is related to the pulse energy entering into the Mach-Zender-Interferometer (MZI) by $U_0 = 2 \cdot 4\pi\epsilon_0 c n_{\text{p}} S_{\text{eff,p}} \int_0^\infty \mathrm{d}\omega\, |\mathcal{E}_{\text{p}}(\omega)|^2$. The factor of 2 takes into account, that the initial pulse energy U_0 is distributed equally into the two arms of the MZI.

Inside the lithium-niobate waveguide, a nonlinear polarization is induced by the mixing of the THz field and the probe field [1]:

$$P^{(2)}(\vec{r},t) = 2\varepsilon_0 \chi_{33} E_{\text{p}}(\vec{r},t) E_{\text{gap}}(\vec{r},t+\tau). \tag{5}$$

τ accounts for the delay we can introduce on the THz arriving at the device but also for timing of the probe pulses reaching the antennas on the two different arms. Any other possible nonlinear mixing processes are ignored here. Mixing of two frequencies within the probe spectrum is not supported by phase-matching and the mixing within the THz spectrum will not generate a detectable signal. In frequency domain the nonlinear polarization reads:

$$P^{(2)}(\vec{r},\omega) = 2\varepsilon_0 \chi_{33} \int \mathrm{d}\Omega \mathrm{e}^{-i\Omega\tau} E_{\text{p}}(\vec{r},\omega-\Omega) E_{\text{gap}}(\vec{r},\Omega). \tag{6}$$

The nonlinear wave equation for the spectral field amplitude in paraxial approximation and slowly varying envelop approximation reads [1]:

$$\frac{\partial}{\partial y} E^{(2)}(\vec{r},\omega) = \frac{i\omega}{2\epsilon_0 c n_{\text{p}}} P^{(2)}(\vec{r},\omega). \tag{7}$$

After integrating along the interaction length l, the nonlinear spectral field at the end plane of the interaction region $\vec{r}_l$ reads:

$$E^{(2)}(\vec{r}_l,\omega) = i\frac{\omega\chi_{33}}{n_{\text{p}} c} \int \mathrm{d}\Omega \mathrm{e}^{-i\Omega\tau} \int_{-\frac{l}{2}}^{\frac{l}{2}} \mathrm{d}y \mathrm{e}^{-ik(\omega)y} E_{\text{p}}(\vec{r},\omega-\Omega) E_{\text{gap}}(\vec{r},\Omega). \tag{8}$$

We assume the input fields $E_\mathrm{p}(\vec{r},\omega-\Omega)$ and $E_\mathrm{gap}(\vec{r},\Omega)$ remain unaffected within the process due to a weak nonlinear interaction. Now we can separate the spectral fields into their spectral and spatial dependencies:

$$E^{(2)}(\vec{r}_l,\omega) = i\frac{\omega\chi_{33}}{n_\mathrm{p}c}\; u_\mathrm{p}(\vec{r}_\perp)u_\mathrm{THz}(\vec{r}_\perp)\int \mathrm{d}\Omega \mathrm{e}^{-i\Omega\tau}\mathcal{E}_\mathrm{p}(\omega-\Omega)\mathcal{E}_\mathrm{gap}(\Omega)\int_{-\frac{l}{2}}^{\frac{l}{2}}\mathrm{d}y \mathrm{e}^{i(k(\omega-\Omega)-k(\omega))y} \tag{9}$$

$$= i\frac{\omega\chi_{33}}{n_\mathrm{p}c}\; u_\mathrm{p}(\vec{r}_\perp)u_\mathrm{THz}(\vec{r}_\perp)\int \mathrm{d}\Omega \mathrm{e}^{-i\Omega\tau}\mathcal{E}_\mathrm{p}(\omega-\Omega)\mathcal{E}_\mathrm{gap}(\Omega)l_\mathrm{eff}(\Omega). \tag{10}$$

with the effective interaction length $l_\mathrm{eff} = l_\mathrm{gap}\ \mathrm{sinc}\left(\frac{n_\mathrm{g}l_\mathrm{gap}}{2c}\Omega\right)$ with the group refractive index of the probe pulse n_g. The total electric field in the near-infrared after the interaction propagating inside the waveguide can be now described by:

$$E_\mathrm{int}(\vec{r},\omega) = u_\mathrm{p}(\vec{r}_\perp)\mathcal{E}_\mathrm{p}(\omega)\left(1+i\Phi(\vec{r}_\perp,\omega)\right)\mathrm{e}^{ik(\omega)y} \tag{11}$$

$$= E_\mathrm{p}(\vec{r},\omega)\left(1+i\Phi(\vec{r}_\perp,\omega)\right), \tag{12}$$

with $\Phi(\vec{r}_\perp,\omega) = \frac{\omega\chi_{33}}{n_\mathrm{p}c}u_\mathrm{THz}(\vec{r}_\perp)\int \mathrm{d}\Omega \mathrm{e}^{-i\Omega\tau}l_\mathrm{eff}(\Omega)\mathcal{E}_\mathrm{p}(\omega-\Omega)\mathcal{E}_\mathrm{gap}(\Omega)$.

1.1.2 Transmission through the MZI

At the end of the MZI two electric probe fields $E_\mathrm{int,1}(\vec{r}_\perp,\omega)$ and $E_\mathrm{int,2}(\vec{r},\omega)$ are combined, both have interacted with the THz field but with a different time-delay $\tau_1 = \tau + \frac{n_\mathrm{g}\Delta L}{2c}$ and $\tau_2 = \tau - \frac{n_\mathrm{g}\Delta L}{2c}$, where the shift in time is determined by the displacement of the two antennas ΔL along the interferometer arms. Additionally there is a phase-offset between the two arms, which is independent on the THz field but origins only from slightly different path-length δd of the two arms:

$$\varphi_\mathrm{MZI} = \frac{n_\mathrm{p}\omega_p}{c}\delta d. \tag{13}$$

The electric field at the output of the MZI reads:

$$E_\mathrm{MZI}(\vec{r},\omega) = \frac{E_\mathrm{p}(\vec{r},\omega)}{\sqrt{2}}\left(1+i\Phi_{\tau_1}(\vec{r}_\perp,\omega)\right)\mathrm{e}^{i\frac{\varphi_\mathrm{MZI}}{2}} + \frac{E_\mathrm{p}(\vec{r},\omega)}{\sqrt{2}}\left(1+i\Phi_{\tau_2}(\vec{r}_\perp,\omega)\right)\mathrm{e}^{-i\frac{\varphi_\mathrm{MZI}}{2}} \tag{14}$$

The pulse energy of the probe signal after the interferometer for a certain time delay τ between the THz and probe field is given by:

$$U_\mathrm{MZI}(\tau) = 4\pi\varepsilon_0 c n_\mathrm{p}\int \mathrm{d}^2r_\perp\int_0^\infty \mathrm{d}\omega|E_\mathrm{MZI}(\vec{r},\omega)|^2 \tag{15}$$

$$= 4\pi\varepsilon_0 c n_\mathrm{p}\int \mathrm{d}^2r_\perp|u_\mathrm{p}(\omega)|^2\int_0^\infty \mathrm{d}\omega|\mathcal{E}_\mathrm{p}(\omega)|^2 \tag{16}$$

$$\left(1+\cos\varphi_\mathrm{MZI} + \frac{i}{2}(1+\mathrm{e}^{i\varphi_\mathrm{MZI}})\left(\Phi_{\tau_1}(\vec{r}_\perp,\omega)-\Phi^*_{\tau_2}(\vec{r}_\perp,\omega)\right) + \frac{i}{2}(1+\mathrm{e}^{-i\varphi_\mathrm{MZI}})\left(\Phi_{\tau_2}(\vec{r}_\perp,\omega)-\Phi^*_{\tau_1}(\vec{r}_\perp,\omega)\right)\right). \tag{17}$$

Terms quadratic in the nonlinear field, i.e. terms proportional to $|\Phi_{\tau_1}(\vec{r}_\perp,\omega)|^2$, $|\Phi_{\tau_2}(\vec{r}_\perp,\omega)|^2$, $\Phi^*_{\tau_1}(\vec{r}_\perp,\omega)\Phi_{\tau_2}(\vec{r}_\perp,\omega)$ and $\Phi^*_{\tau_2}(\vec{r}_\perp,\omega)\Phi_{\tau_1}(\vec{r}_\perp,\omega)$ have been neglected, since we assume a weak interaction. Now we reinsert the expression of Φ and U_0 and define $\delta\tau = \frac{n_\mathrm{g}\Delta L}{2c}$:

$$U_\mathrm{MZI}(\tau) = \frac{U_0}{2}\left(1+\cos\varphi_\mathrm{MZI}\right) + 2i\pi\varepsilon_0\chi_{33}\int \mathrm{d}^2r_\perp|u_\mathrm{p}(\vec{r}_\perp)|^2u_\mathrm{THz}(\vec{r}_\perp)\int \mathrm{d}\Omega\int_0^\infty \mathrm{d}\omega\,\omega \tag{18}$$

$$\Big[(1+\mathrm{e}^{i\varphi_\mathrm{MZI}})\left(\mathcal{E}^*_\mathrm{p}(\omega)\mathcal{E}_\mathrm{p}(\omega-\Omega)\mathcal{E}_\mathrm{gap}(\Omega)\mathrm{e}^{-i\Omega(\tau+\delta\tau)} - \mathcal{E}_\mathrm{p}(\omega)\mathcal{E}^*_\mathrm{p}(\omega-\Omega)\mathcal{E}^*_\mathrm{gap}(\Omega)\mathrm{e}^{i\Omega(\tau-\delta\tau)}\right) \tag{19}$$

$$+ (1+\mathrm{e}^{-i\varphi_\mathrm{MZI}})\left(\mathcal{E}^*_\mathrm{p}(\omega)\mathcal{E}_\mathrm{p}(\omega-\Omega)\mathcal{E}_\mathrm{gap}(\Omega)\mathrm{e}^{-i\Omega(\tau-\delta\tau)} - \mathcal{E}_\mathrm{p}(\omega)\mathcal{E}^*_\mathrm{p}(\omega-\Omega)\mathcal{E}^*_\mathrm{gap}(\Omega)\mathrm{e}^{i\Omega(\tau+\delta\tau)}\right)\Big]. \tag{20}$$

We define now the spectral auto-correlation of the probe field as:

$$C_{\mathrm{p}}(\Omega)=\frac{\int_0^\infty \mathrm{d}\omega\omega\mathcal{E}_{\mathrm{p}}^*(\omega)\mathcal{E}_{\mathrm{p}}(\omega-\Omega)}{\int_0^\infty \mathrm{d}\omega\omega|\mathcal{E}_{\mathrm{p}}(\omega)|^2} \tag{21}$$

and since we are using a narrow probe spectrum (bandwidth $\delta\omega \ll \omega_{\mathrm{p}}$), we can assume $C_{\mathrm{p}}^*(\Omega)=C_{\mathrm{p}}(\Omega)$ and $\int_0^\infty \mathrm{d}\omega\omega|\mathcal{E}_{\mathrm{p}}(\omega)|^2 \approx \omega_{\mathrm{p}}\int_0^\infty \mathrm{d}\omega|\mathcal{E}_{\mathrm{p}}(\omega)|^2$. Furthermore we define the overlap factor $\Gamma=\frac{\int_{\mathrm{LN}}\mathrm{d}^2 r_\perp |u_{\mathrm{p}}(\vec{r}_\perp)|^2 u_{\mathrm{THz}}(\vec{r}_\perp)}{\int \mathrm{d}^2 r_\perp |u_{\mathrm{p}}(\vec{r}_\perp)|^2}=\frac{\int_{\mathrm{LN}}\mathrm{d}^2 r_\perp |u_{\mathrm{p}}(\vec{r}_\perp)|^2 u_{\mathrm{THz}}(\vec{r}_\perp)}{S_{\mathrm{eff,p}}}$.

Using those definitions, the pulse energy simplifies to:

$$U_{\mathrm{MZI}}(\tau)=\frac{U_0}{2}\left(1+\cos\varphi_{\mathrm{MZI}}\right)+2i\pi\varepsilon_0\omega_{\mathrm{p}}\chi_{33}\Gamma S_{\mathrm{eff,p}}\int \mathrm{d}\Omega C_{\mathrm{p}}(\Omega)\int_0^\infty \mathrm{d}\omega\ |\mathcal{E}_{\mathrm{p}}(\omega)|^2 \tag{22}$$

$$\left[\mathcal{E}_{\mathrm{gap}}(\Omega)\mathrm{e}^{-i\Omega\tau}-\mathcal{E}_{\mathrm{gap}}^*(\Omega)\mathrm{e}^{i\Omega\tau}\right]\left[\mathrm{e}^{i\Omega\delta\tau}(1+\mathrm{e}^{i\varphi_{\mathrm{MZI}}})+\mathrm{e}^{-i\Omega\delta\tau}(1+\mathrm{e}^{-i\varphi_{\mathrm{MZI}}})\right]. \tag{23}$$

Separating the integration over Ω into positive and negative frequencies and using the fact, that $C_{\mathrm{p}}(-\Omega)=C_{\mathrm{p}}^*(\Omega)=C_{\mathrm{p}}(\Omega)$, $l_{\mathrm{eff,p}}(-\Omega)=l_{\mathrm{eff}}(\Omega)$ and $\mathcal{E}_{\mathrm{gap}}(-\Omega)=\mathcal{E}_{\mathrm{gap}}^*(\Omega)$, we get:

$$U_{\mathrm{MZI}}(\tau)=\frac{U_0}{2}\left(1+\cos\varphi_{\mathrm{MZI}}\right)-2i\sin\varphi_{\mathrm{MZI}}\cdot\frac{\omega_{\mathrm{p}}\chi_{33}}{n_{\mathrm{p}}c}\Gamma S_{\mathrm{eff,p}}\int_0^\infty \mathrm{d}\Omega C_{\mathrm{p}}(\Omega) \tag{24}$$

$$\sin(\Omega\delta\tau)\left(\mathcal{E}_{\mathrm{gap}}(\Omega)\mathrm{e}^{-i\Omega\tau}-\mathcal{E}_{\mathrm{gap}}^*(\Omega)\mathrm{e}^{i\Omega\tau}\right). \tag{25}$$

Finally, we define the frequency-dependent sensitivity of the detector $R(\Omega)=2i\sin(\Omega\tau)\,\mathrm{sinc}\left(\frac{n_{\mathrm{g}}l_{\mathrm{gap}}}{2c}\Omega\right)C_{\mathrm{p}}(\Omega)G(\Omega)$ and we combine back the integration over positive and negative Ω:

$$U_{\mathrm{MZI}}(\tau)=\frac{U_0}{2}\left(1+\cos\varphi_{\mathrm{MZI}}-\sin\varphi_{\mathrm{MZI}}\ g_{\mathrm{eo}}\Gamma\int \mathrm{d}\Omega \mathrm{e}^{-i\Omega\tau}R(\Omega)E_{\mathrm{THz}}(\Omega)\right), \tag{26}$$

$$\tag{27}$$

where we induced the electro-optic coupling factor $g_{\mathrm{eo}}=\frac{\omega_{\mathrm{p}}\chi_{33}}{n_{\mathrm{p}}c}l_{\mathrm{gap}}=-\frac{n_{\mathrm{p}}^3\omega_{\mathrm{p}}r_{33}}{2c}l_{\mathrm{gap}}$.

2 Supplementary Note 2 (Simulations)

2.1 THz field inside antenna gap

We used the commercial simulation software CST Microwave Studio to simulate the THz field inside the antenna gap for a incoming plane wave. To avoid long calculation times due to large computation times due to a large simulation volume, the substrate thickness is reduced from 500 µm in reality to 30 µm in the simulation.

To obtain the spatial profile of the THz field inside the antenna gap, we use the frequency-domain solver at the resonance frequency of the antenna design and evaluate the field along the cross-section of the waveguide with the field monitor in the center of the antenna gap (see also Fig. 1 of the main text). The spatial mode profile $u_{\mathrm{THz}}(\vec{r}_\perp)$ is normalized, so that $\int \mathrm{d}^2 r_\perp |u_{\mathrm{THz}}(\vec{r}_\perp)|^2=S_{\mathrm{eff,THz}}$, where the effective mode area is in general defined as $S_{\mathrm{eff}}=\frac{\left(\int \mathrm{d}\vec{r}_\perp |E(\vec{r}_\perp)|^2\right)^2}{\int \mathrm{d}\vec{r}_\perp |E(\vec{r}_\perp)|^4}$.

To obtain the frequency-dependent field enhancement $G(\Omega)$ between the free-space THz field E_{THz} and the confined field strength inside the waveguide E_{gap}, the time-domain solver in combination with a plane-wave excitation is used to calculate the field in the center of the waveguide $\mathcal{E}_{\mathrm{sim}}(\Omega)$ (green arrow in Supplementary Fig. 1 a and red cross

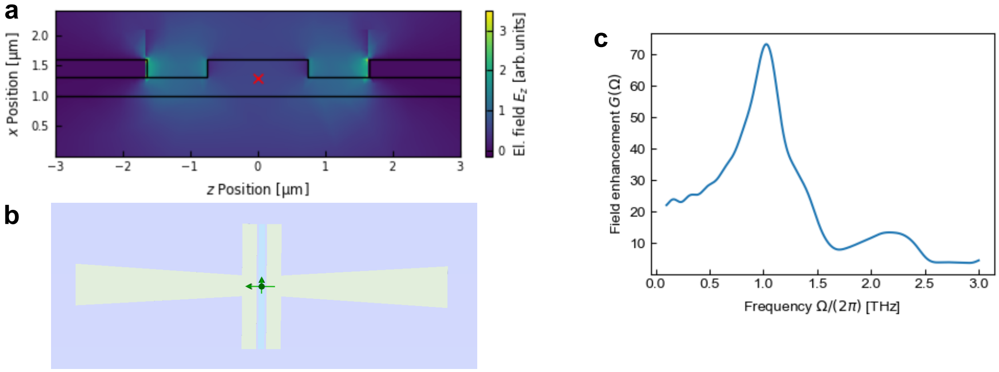

Supplementary Fig. 1: a: Position of CST field probe (green arrows) in the center of the antenna. b: Spatial profile of electric field $u_{\mathrm{THz}}(\vec{r}_\perp)$ and position of the field probe at $\vec{r}_{\mathrm{center}}$ (red cross). c: Exemplary field enhancement simulated in CST with a resonance frequency of 1.02 THz.

in Supplementary Fig. 1 b). We use an identical simulation without the antenna structure to obtain the reference field $\mathcal{E}_{\mathrm{ref}}(\Omega)$. To be consistent with the definition of the spatial field $\varepsilon(\vec{r}_\perp)$, the field enhancement is rescaled with the normalized spatial field in the center of the waveguide $u_{\mathrm{THz}}(\vec{r}_{\mathrm{center}})$:

$$G(\Omega) = \frac{1}{u_{\mathrm{THz}}(\vec{r}_{\mathrm{center}})} \cdot \frac{\mathcal{E}_{\mathrm{sim}}(\Omega)}{\mathcal{E}_{\mathrm{ref}}(\Omega)} \approx 1.7 \cdot \frac{\mathcal{E}_{\mathrm{sim}}(\Omega)}{\mathcal{E}_{\mathrm{ref}}(\Omega)}. \tag{28}$$

In Supplementary Fig. 1 a the obtained field enhancement for an antenna with a arm length of 40 µm is shown. The design corresponds to the device investigated in Fig. 3 of the main text. The resonance frequency of the antenna designs, as given in Tab. 1 of this document correspond to the frequency of maximum field enhancement $G(2\pi \cdot \nu_{\mathrm{res}}) = G_{\max}$.

2.2 Overlap factor inside antenna gap

We are performing a finite element simulation using COMSOL Wave Optics to describe the near-infrared probe mode inside the lithium niobate ridge waveguide. We determined a group refractive index of $n_{\mathrm{g}} = 2.3$ at the probe wavelength of 1575 nm and the spatial field profile $u_{\mathrm{p}}(\vec{r}_\perp)$ inside the antenna gap. The energy density as plotted in Fig. 1 of the main text is given by $|u_{\mathrm{p}}(\vec{r}_\perp)|^2$ and we use the normalization $\int \mathrm{d}^2 r_\perp |u_{\mathrm{p}}(\vec{r}_\perp)|^2 = S_{\mathrm{eff,p}}$.
In combination with the simulation of the THz field (Supplementary Information Note 2.1), we can calculate the overlap factor between the probe and THz field and we find $\Gamma = 0.54$.

2.3 Frequency response

We have seen the spectral sensitivity $R(\Omega)$ of the detector is determined by a combination of different design parameters, but also by the temporal and spectral characteristics of the probe signal (Supplementary Fig. 2 a). The different influences are separated in four individual frequency-dependent functions:

$$R(\Omega) = C_{\mathrm{p}}(\Omega) \cdot P(\Omega) \cdot D_{\mathrm{MZI}}(\Omega) \cdot G_{\mathrm{ant}}(\Omega). \tag{29}$$

The design of the antenna and the resulting resonance is expressed in the frequency dependent field enhancement $G_{\mathrm{ant}}(\Omega)$, which has a strong influence on the spectral response and can be tuned to the frequency range required for the particular application. In Supplementary Fig. 2 a (red line) the field enhancement for an bow-tie antenna optimized for a frequency of 1 THz is presented, as it has been obtained in a finite element simulation in CST (Supplementary Information 2.1). In addition, the antenna geometry defines the interaction region and influences

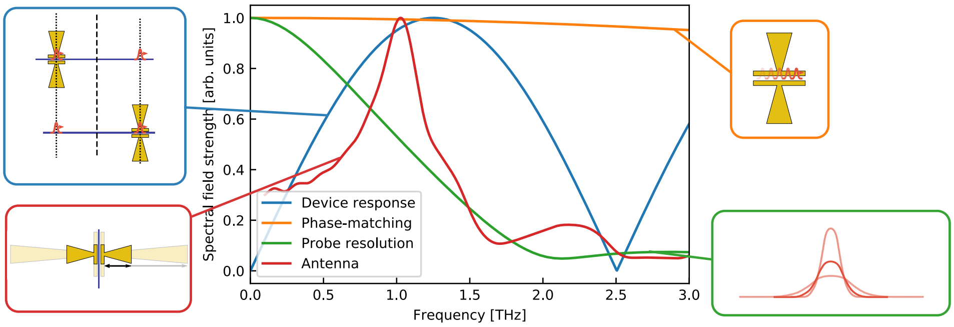

Supplementary Fig. 2: Expected frequency response of the thin-film lithium niobate THz detectors: The push-pull-configuration implemented by displacing the antennas along the waveguide arms (blue box upper left) introduces a sin-factor and the optimum corresponds to the frequency, where half a period time matches the time of propagating between the two antennas. The phase-matching between the probe pulses propagating along the antenna gap (orange box, upper right), and the confined THz field leads to a reduced response for increasing THz frequency. The antenna design determines the field enhancement of the THz, which is here simulated for a antenna at 1 THz. The duration of the probe pulses determines the upper frequency-limit, that can be still resolved. c: THz spectrum retrieved Fourier transforming the measured trace shown in b compared to the expected frequency response of this devices.

the observed spectrum due to the phase matching $P(\Omega)$ between the probe pulses propagating in the gap with an effective group index n_g and a length of l_gap and the THz field:

$$P(\Omega) = \mathrm{sinc}\left(\frac{n_\mathrm{g} l_\mathrm{gap}}{2c}\Omega\right). \tag{30}$$

Even though also the THz propagates from the center of the waveguide to the two ends of the interaction region, we assume for simplicity a constant spatial phase of the THz field in the description of the phase-matching, since the effects of co- and counter-propagation for half of the interaction compensate for each other. For the antenna geometries investigated in the current study the gap length is always short enough to ensure the phase-matching has only minor influence onto the detected frequencies (Supplementary Fig. 2 a, orange line) compared to the field enhancement of the antenna (Supplementary Fig. 2 a, red line). Since the spatial displacement of the antennas along the waveguides is optimized for one particular THz frequencies, other frequencies are detected less efficient resulting in another frequency-dependent term:

$$D_\mathrm{shift}(\Omega) = 2i\sin\left(\frac{n_\mathrm{g}\Delta L}{2c}\Omega\right). \tag{31}$$

The factor of i takes account for the fact, that the probes are interacting with the electric field at two points in time $t = \tau \pm \Delta\tau$ symmetrically around the delay-time τ and the modulation is given by the difference of the field strength in these two points. As a result the measured trace follows the derivative of the incoming THz waveform spectrally filtered with the sin-function and we observe a phase-shift of $\frac{\pi}{2}$ between the incoming signal and the detected waveform (compare Fig. 1 in the main text). The maximum of the sinus-function is centered at the designed frequency, so that its influence on the detected spectral range is small compared to the antenna resonance. Finally also the properties of the probe pulses influence the spectral bandwidth of the detected THz frequencies. To resolve the THz frequencies in time-domain, the probe pulses need to be shorter than at least half the period time of the THz signal. In the frequency-dependent response $R(\Omega)$ this fact is expressed in the normalized auto-correlation of the complex spectral

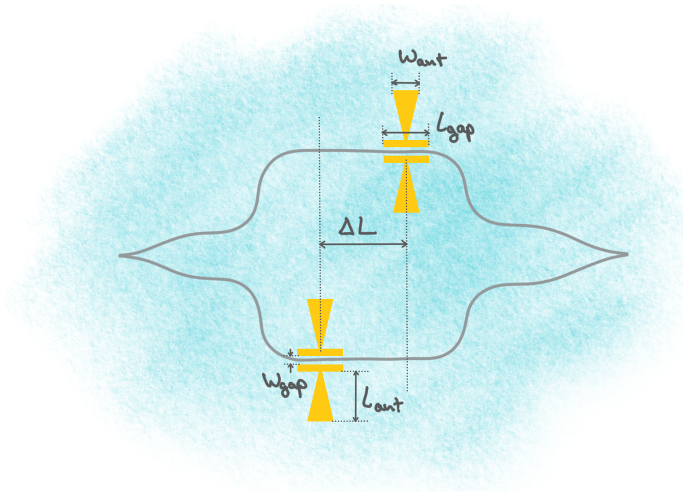

Supplementary Fig. 3: Sketch of a integrated detector indicating the dimensions varying between different devices.

probe field $\mathcal{E}_\mathrm{p}(\omega)$:

$$C_\mathrm{p}(\Omega) = \frac{\int_0^\infty \mathrm{d}\omega\, \mathcal{E}_\mathrm{p}^*(\omega)\mathcal{E}_\mathrm{p}(\omega-\Omega)}{\int_0^\infty \mathrm{d}\omega\, |\mathcal{E}_\mathrm{p}(\omega)|^2}. \tag{32}$$

Due to dispersion and nonlinear effects inside the fiber guiding the probe signal from the laser to the chip and the on-chip waveguide, as well as the frequency-dependent coupling at the grating couplers, the true pulse-length of the probe-signal inside the antenna gap is hard to predict. For a first estimation of the spectral auto-correlation term we use the spectrum measured after out-coupling from the chip and add the expected group-delay dispersion of the 1.1 m long fiber leading to a estimated pulse duration of 400 fs. The obtained spectral auto-correlation (Supplementary Fig. 1 green line) reduces by one half at a frequency of 1.2 THz, and therefore is the dominant limitation in detectable frequency. However, the latter is only a rough estimation without taking into account the self-phase modulation inside the fiber, which could reduce the true pulse length. Furthermore the pulse duration is not a fundamental limitation of the presented devices but rather given by the laser source and the current experimental configuration.

3 Supplementary Note 3 (Experiment)

3.1 Device dimensions

For the devices shown in this study, bow-tie antennas of different dimensions are used. All parameters differing between different devices are indicated in Fig. 3 and listed in Tab. 1 for the devices investigated in the main text. All other dimensions remained unchanged and are given in the Supplementary material of reference [2].

3.2 Setup

The experimental setup used to characterize the integrated THz detectors is presented in Supplementary Fig. 4.

3.3 Emission of photoconductive antenna

The THz spectrum emitted by the photoconductive antenna has been characterized by electro-optic sampling in a ⟨110⟩-cut zinc telluride crystal of 200 µm thickness. In Supplementary Fig. 5 the detected free space THz waveform (left) and the corresponding spectral THz field (right) is plotted. This signal was used as input THz signal to calculate the expected signal using the thin-film lithium niobate detection scheme.

Type	l_{arm} [µm]	w_{arm} [µm]	l_{gap} [µm]	ΔL [µm]	w_{gap} [µm]	ν_{res} [GHz]	Figure
MZI	40	10	7.5	52	3.0	1028	2 a & b, 3
MZI	90	30	30	119	3.0	414	2 b
MZI	200	30	30	215	3.0	216	2 b
MZI	90	30	45	128	2.7	370	4
Single	40	30	20	–	3.3	864	5
MZI	40	10	22.5	57	3.3	892	5

Supplementary Table 1: Dimensions of different antennas. The different parameters are indicated in Fig. 3. The fist column indicates, whether the device is based on a interferometer structure (MZI) or a single antenna (Single). The resonance frequency of the antenna ν_{res} is given by the peak frequency of simulated the field enhancement $G(\Omega)$ (see Supplementary material section 2.1). The last column gives the number of the figures showing measurements using the particular device in the main text.

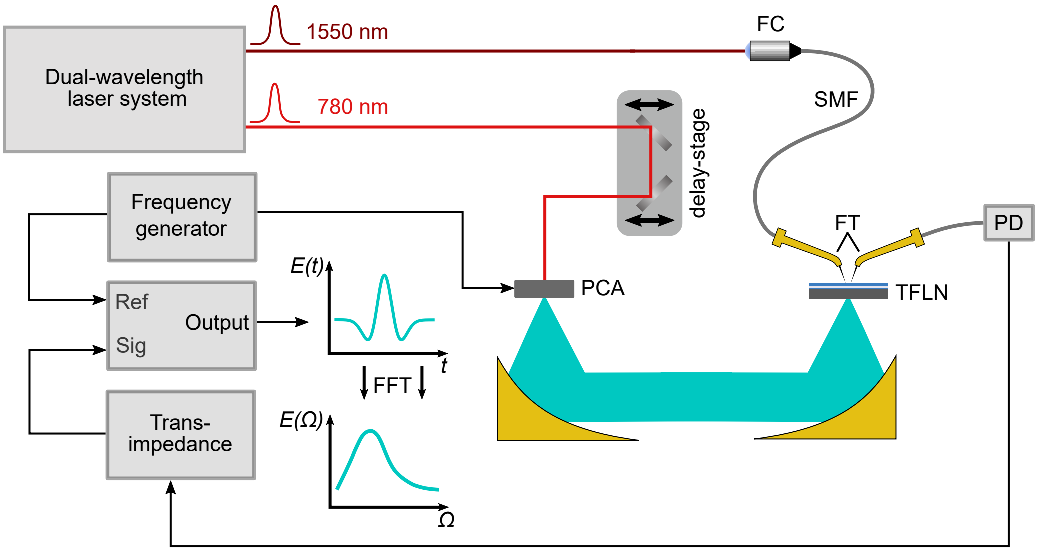

Supplementary Fig. 4: We are using a dual-wavelength laser system provided by Menolo Systems emitting femtosecond pulses origin from a erbium doped fiber oscillator at 1560 nm (dark red line) and its second harmonic at 780 nm (light red line) at a repetition rate of 60 MHz. The second harmonic signal acts as pump for a photo-conductive antenna (PCA). To enable a lock-in detection of the THz-radiation the bias of the photo-conductive antenna is modulated with a 90 kHz rectangular signal. The emitted THz field (cyan area) is collected and focused onto the integrated device by a pair of parabolic mirrors with 1:1 imaging. Integrated grating coupler is used to couple the probe signal at 1560 nm emitted from the cleaved facet of a single mode fiber into the on-chip waveguide and back into a fiber after passing through the integrated device. Two piezo-stages provided by SmarAct control the position of the fiber tips (FT) on top of the grating couplers with a distance of 10 µm and align the in-plane position automatically to maximum transmission through the chip before the measurement is started. The output fiber directs the probe signal to an InGaAs photo-diode (PD) and the photo-current is amplified by an external trans-impedance gain before being measured with an lock-in amplifier at 90 kHz. To sample along the THz field in time-domain, a stage on the pump-line controls the time-delay between the THz signal and the probe pulses arriving at the detection device.

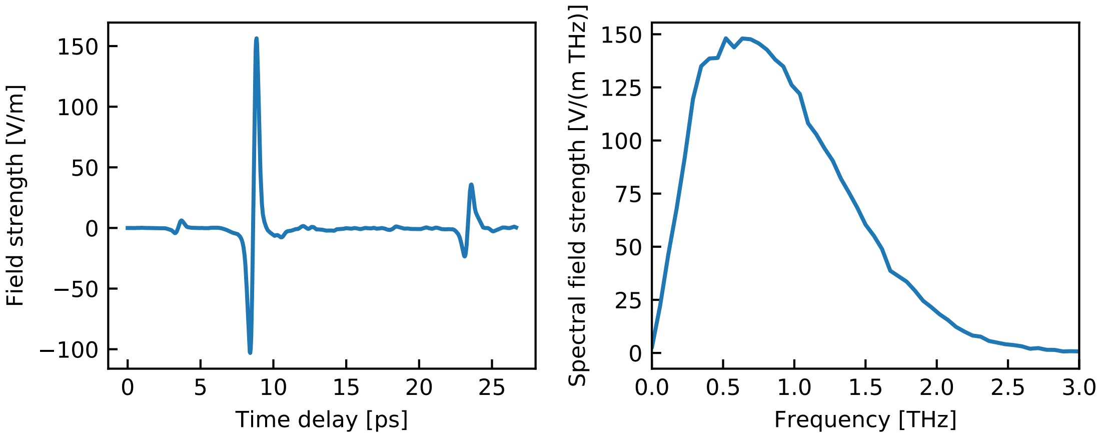

Supplementary Fig. 5: THz waveform in time (left) and corresponding spectral field (right) of the photoconductive antenna utilized within the study measured with electro-optic sampling using a 200-µm zinc telluride crystal.

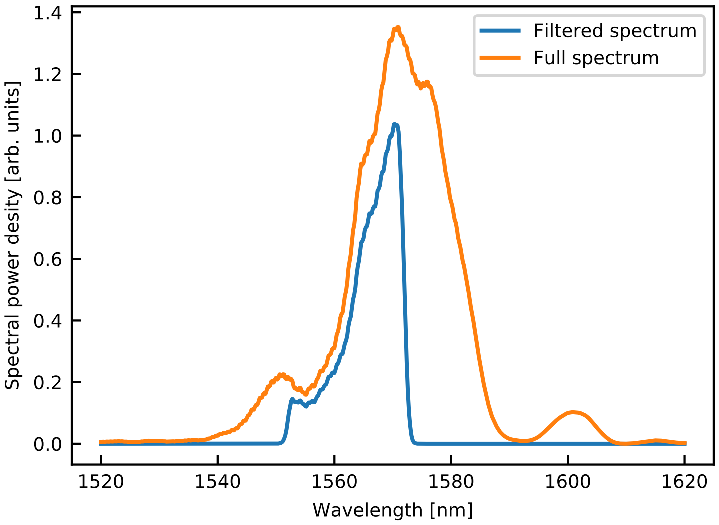

Supplementary Fig. 6: Spectral intensity of probe signal before reaching the detector with (blue solid line) and without (orange solid line) short-pass filter.

3.4 Spectral filtering of the probe signal

For the detection with a single antenna device, the probe signal was spectrally filtered after the interaction with the THz radiation. A fiber-coupled adjustable bandpass filter was placed between the fiber tip collecting the probe signal from the chip and the photo-detector. As shown in Supplementary Fig. 6, the low-frequency (long-wavelength) edge of the filter was placed in the center of the probe signal, so that it is acting as a short-pass filter.

3.5 Operation point

Within out study we measured the emission of the photo-conductive antenna with 10 different interferometric devices under the same conditions. The antennas printed onto these devices have an arm length of 90 µm but differ in their gap length and width. In Supplementary Fig. 7, the ratio between the detected signal at the resonance frequency and the one simulated for the particular device dimensions is plotted for each device as a horizontal dotted line, which range from 40 % to 2.5 times the expected value. Additionally the influence of the operation point onto the signal

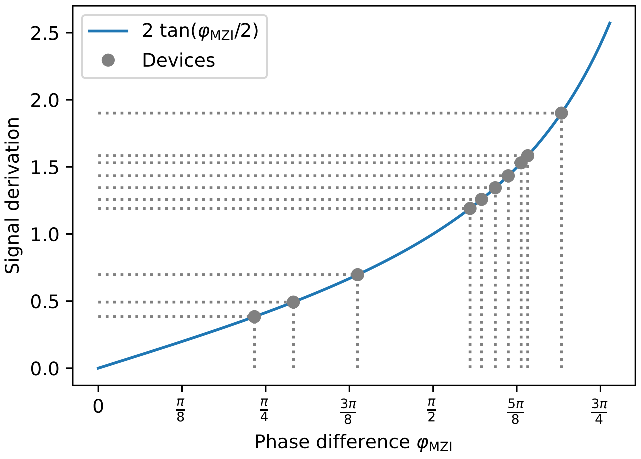

Supplementary Fig. 7: Response of the interferometric devices depending on the operation point of the interferometer φ_{MZI}. The relative change of the signal depending on the operation point is plotted in blue. Additionally the ratio between the signal observed with 10 different devices and the simulated signal is plotted over the operation point leading to the observed deviation is plotted in gray dots.

$\frac{\sin\varphi_{\mathrm{MZI}}}{1+\cos\varphi_{\mathrm{MZI}}} = 2\tan(\frac{\varphi_{\mathrm{MZI}}}{2})$ is plotted.

Supplementary References

[1] P. E. Powers and J. W. Haus. "Fundamentals of Nonlinear Optics." Fundamentals of Nonlinear Optics, Second Edition (2017).

[2] A. Herter, A. Shams-Ansari, F. F. Settembrini, H. K. Warner, J. Faist, M. Lončar, and I. C. Benea-Chelmus. "Terahertz waveform synthesis in integrated thin-film lithium niobate platform." Nature Communications 2023 14:1, **14**:1–9 (2023).
